# LncRNA AC079630.4 expression associated with the progression and prognosis in lung cancer

**DOI:** 10.18632/aging.203310

**Published:** 2021-07-19

**Authors:** Li-Fang Wang, Li-Ping Wu, Jian-Dong Wen

**Affiliations:** 1 Drug Clinical Trial Office, Ganzhou People's Hospital, Ganzhou 341000, China

**Keywords:** lncRNA, AC079630.4, lung cancer, progression, prognosis

## Abstract

Mounting evidence has demonstrated the important role of long non-coding RNAs (lncRNAs) in the development and progression of lung cancer. In this study, we combined the methods of bioinformatics analysis and experimental validation, and aim to investigate the clinical significance and underlying mechanism of the novel lncRNA AC079630.4 in lung cancer. Finally, we found that AC079630.4 was significantly down-regulated in lung cancer tissues, including in its subtypes. Samples with low AC079630.4 expression had a more advanced pathological stage and a worse prognosis than those with high expression. In functional prediction, the KEGG pathway of apoptosis and the TRAIL signaling pathway were enriched in the samples with high AC079630.4 expression. In experimental validation, AC079630.4 over-expression could significantly inhibit the proliferation and clonality, and up-regulated the receptors of TRAIL (TRAIL-R1 and TRAIL-R2) in lung cancer cells. In conclusion, we adopted the methods of bioinformatics analysis and experimental validation, and identified a novel lncRNA of AC079630.4 as a tumor suppressor in lung cancer.

## INTRODUCTION

Lung cancer is one of the most common malignancies and a prominent cause of cancer deaths worldwide [1]. Among the lung cancer cases, non-small cell lung cancer (NSCLC) accounted for more than 80%, including the subtypes of adenocarcinoma, squamous cell carcinoma and large cell carcinoma [2]. Despite great improvement in the therapies, the 5-year survival rate is still at a very low level of less than 20% [3]. Thus, it is important to explore novel diagnostic and therapeutic biomarkers.

Long non-coding RNAs (lncRNA) were defined as a class of non-coding transcripts longer than 200 nucleotides in length [4]. Usually, lncRNAs were considered non-functional, but recent studies indicated a critical role of several lncRNAs in the initiation, progression, and prognosis of various tumors [5]. For example, linc01833 could function as a competitive endogenous RNA (ceRNA) to adsorb miR-519e-3p through a sponge and regulate S100A4 in lung cancer, and thereby get involved in the progression of lung adenocarcinoma [6]. LncRNA ZFPM2-AS1 promoted the proliferation, migration, and invasion of NSCLC cells via the JAK-STAT and AKT pathways [7]. However, the expression and underlying mechanism of lncRNA AC079630.4 in lung cancer remain unclear.

In this study, we adopted the novel method of probe re-annotation, and found a down-regulation of AC079630.4 in lung cancer. The down-regulated AC079630.4 expression levels were associated with a more advanced pathological stage and a worse prognosis. AC079630.4 over-expression was also demonstrated to inhibit lung cancer cell proliferation and clonality, and regulate the apoptosis signaling pathway.

## MATERIALS AND METHODS

### Data and sample collection

Microarray data of GSE19188 (n=156), GSE31210 (n=226) and GSE30219 (n=293) and related clinicopathological features were downloaded from the database of Gene Expression Omnibus (GEO) (https://www.ncbi.nlm.nih.gov/geo/). All datasets were originated from the GPL570 microarray platform ([HG-U133_Plus_2] Affymetrix Human Genome U133 Plus 2.0 Array). The dataset GSE19188 contained a total of 91 tumor- and 65 adjacent normal lung tissue samples, including the NSCLC subtypes of adenocarcinoma (ADC), large cell carcinoma (LCC) and squamous cell carcinoma (SCC). The dataset GSE31210 contained 226 samples of pathological stage I-II ADC and 20 adjacent normal tissue samples. The dataset GSE30219 contained 293 lung cancer samples.

Furthermore, 10 pairs of lung cancer and adjacent normal tissues (surgical resection) were collected and frozen at -70° C. This study was funded by Science and Technology Plan Project of Jiangxi Provincial Health and Family Planning Commission (20177255).

### Probe re-annotation

**Figure 1:**
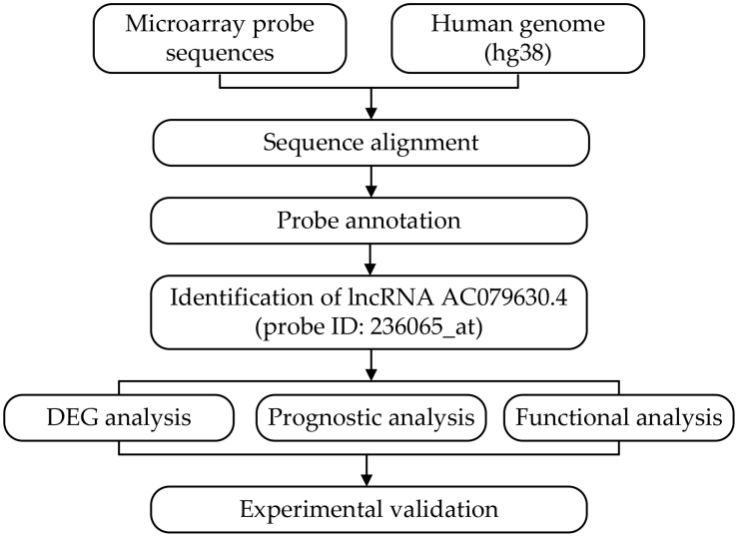
**Flowchart of the whole procedures.** DEG, differentially expressed gene.

**Figure 2:**
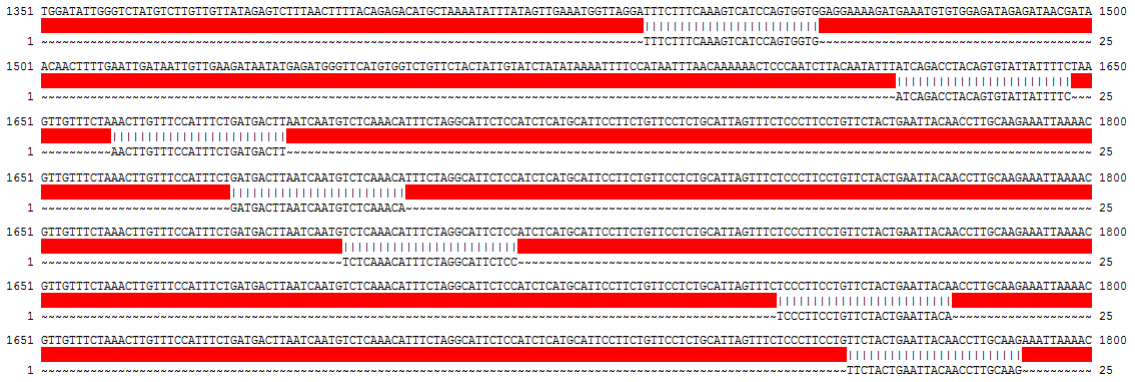
List of the probes matched to AC079630.4 in the probe-set of 236065_at.

In the Affymetrix microarray platform, a set of 11~22 probes represented each gene. However, much information about the long non-coding RNAs (lncRNA) was covered with the update of the human genome and annotation files. Thus, we discarded the original probe-set definitions, and re-annotated the probe sets by mapping each probe via their sequence to the reference sequence (hg38) using the previous method [8–10]. Finally, the transcript representing lncRNA AC079630.4 (probe ID: 236065_at) was identified according to the following criteria: (i) detected by more than three probes; (ii) each probe was mapped without mismatch; (iii) each probe was matched to only one transcript in probe-transcript pairs. In the probe-set of 236065_at, seven out of eleven probes could map to AC079630.4 without any mismatch (Figures 1, 2).

### Gene set enrichment analysis (GSEA)

In the datasets of GSE31210 and GSE30219, all samples were divided into two groups according to the median value of AC079630.4 expression. GSEA was performed between the high- and low-expression groups to identify potential function of AC079630.4, and the annotation file of c2.cp.kegg.v5.2.symbols.gmt was chosen as the reference gene set. A false discovery rate (FDR) of less than 0.05 was selected as the cut-off.

### Gene ontology (GO) analysis

To investigate the potential function of AC079630.4, we performed GO analysis of related biological processes on its related genes using the online tool of ToppFun (https://toppgene.cchmc.org/enrichment.jsp). An FDR of less than 0.05 was selected as the cut-off.

### Cell culture and transfection

The human lung cancer cell lines (H1299 and H520) were purchased from Shanghai Institutes of Biochemistry and Cell Biology (Shanghai, China). All cells were cultured in RPMI-1640 (Thermo Fisher, USA) supplemented with 10% bovine serum (Thermo Fisher, USA) in a 5% CO_2_ incubator at 37° C. Then, H1299 and H520 cells were seeded into 6-well plates, and transfected with the AC079630.4 over-expression plasmid (pcDNA3.1-AC079630.4) or the negative control (pcDNA3.1-NC) provided by GenePharma (Shanghai, China) using lipofectamine 2000 reagent according to the manufacturer's protocol (Thermo Fisher, USA).

### RNA extraction and quantitative RT-PCR

Total RNA was extracted using TRIzol reagent (Invitrogen, USA), and then synthesized into first-strand cDNA using a synthesis kit (Thermo Fisher, USA). Gene expression levels were subsequently detected by the Applied Biosystems 7500 real-time PCR system (Thermo Fisher, USA) using the SYBR Green master mix (Thermo Fisher, USA). Relative expression levels were calculated using the 2^−ΔΔCt^ method. Each sample was performed in triplicate.

### Western blot analysis

Total protein was isolated using the extraction kits (BestBio Science, China). The protein samples were separated by SDS-PAGE (Sigma, USA), transferred electrophoretically to the PVDF membrane (Millipore, USA) and blocked with 5% BSA. Then, the membranes were incubated with primary and secondary antibodies (ProScience, USA). Immunoblots were visualized by the enhanced chemiluminescent kits (Sigma, USA). The grey density of β-actin band was used as an internal control to normalize the densities of target proteins.

### Cell proliferation assay

Cells were seeded into 96-well plates at a density of 1x10^5^ cells/well, and incubated at 37° C for 24, 48 and 72h. Subsequently, 10μl solution of Cell Counting Kit-8 (CCK-8) solution (Beyotime, China) was added to each well and incubated for 2h. The absorbance at 450nm was measured in each well using a microplate reader (Bio-Rad, USA).

### Colony forming assay

1000 cells were incubated in 6-well plate for 12 days at 37° C. Then, cells were fixed with 4% paraformaldehyde and stained with 0.1% crystal violet. Colonies were quantified under a light microscope (magnification, x100).

### Statistical analysis

Continuous variables were presented as mean ± standard deviation (SD) and compared using one-way ANOVA analysis. For paired data, paired t test was conducted. Categorical variables were described by percentages and compared via Chi-Squared tests. Cox proportional hazards regression analysis was used to evaluate the association between AC079630.4 expression and the prognosis.

## RESULTS

### Down-regulation of lncRNA AC079630.4 expression in lung cancer and its subtypes

**Figure 3:**
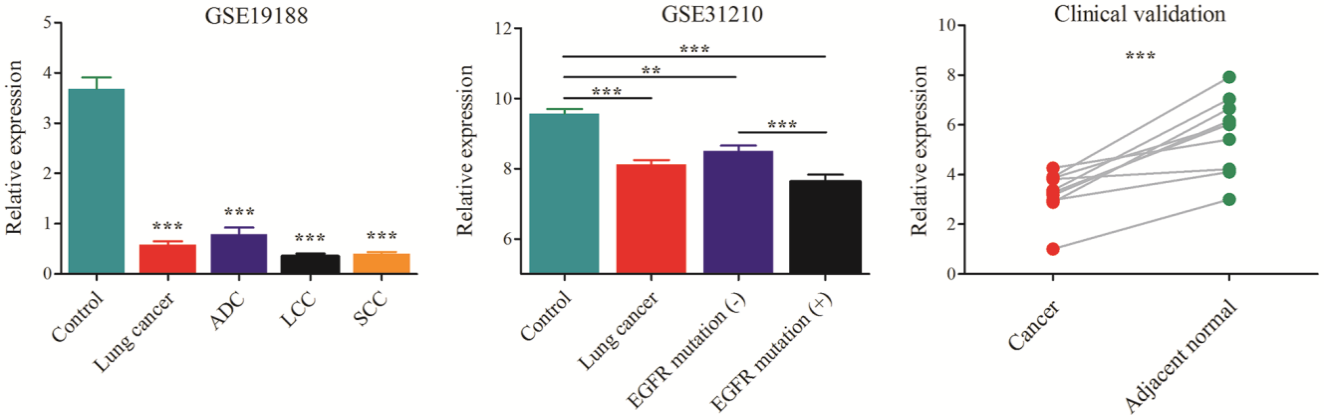
**Down-regulation of AC079630.4 expression in lung cancer and its subtypes.** *P<0.05, **P<0.01, ***P<0.001.

The dataset GSE19188 contained a total of 91 tumor- and 65 adjacent normal lung tissue samples, including the NSCLC subtypes of ADC, LCC and SCC. After probe re-annotation and differentially expressed gene analysis, we identified 169 differentially expressed lncRNAs (Supplementary Figure 1 and Supplementary Table 1). Compared with the adjacent normal tissues, AC079630.4 was significantly down-regulated in lung cancer tissues (Figure 3). When considering the subtypes, it was also lowly expressed in ADC, LCC and SCC. The dataset GSE31210 included 226 samples of pathological stage I-II ADC and 20 adjacent normal tissue samples. AC079630.4 was significantly down-regulated in both the patients with and without EGFR mutation. Moreover, the patients with EGRF mutation showed a lower AC079630.4 expression than those without EGRF mutation. These indicated that AC079630.4 might act as a protective gene in lung cancer. In the clinical validation of 10 paired cancer and adjacent normal tissues, AC079630.4 was also obviously down-regulated in lung cancer.

### Association between AC079630.4 expression and clinicopathological features of lung cancer

The dataset GSE31210 contained 226 samples of pathological stage I-II ADC (Table 1). All samples were divided into two groups according to the median value of AC079630.4 expression. Comparably, patients with high AC079630.4 expression had a better TNM stage (*P*<0.001) and a lower incidence of EGFR mutation (*P*=0.002).

**Table 1:** Analysis of the association between lncRNA AC079630.4 expression and clinicopathological features of lung cancer based on dataset GSE31210.

**Variables**	**Low expression (n=113)**	**High expression (n=113)**	***P* value**
Age (years)	56.0 ± 0.66	59.2 ± 0.73	0.457
Gender			
Male	58 (51.3%)	47 (41.6%)	0.142
Female	55 (48.7%)	66 (58.4%)	
Smoking			
Yes	61 (54.0%)	50 (44.2%)	0.143
No	52 (46.0%)	63 (55.8%)	
TNM stage			
Ia	41 (36.3%)	73 (64.6%)	<0.001
Ib	27 (23.9%)	27 (23.9%)	
II	45 (39.8%)	13 (11.5%)	
ALK fusion			
Yes	7 (6.2%)	4 (3.5%)	0.354
No	106 (93.8%)	109 (96.5%)	
EGFR mutation			
Yes	61 (54.0%)	38 (33.6%)	0.002
No	52 (46.0%)	75 (66.4%)	
KRAS mutation			
Yes	13 (11.5%)	7 (7.1%)	0.160
No	100 (88.5%)	106 (92.9%)	
MYC expression			
High	8 (7.1%)	9 (8.0%)	0.801
Low	104 (92.9%)	103 (92.0%)	

The dataset GSE30219 contained 293 lung cancer samples (Table 2). Comparably, patients with high AC079630.4 expression had a better T stage (*P*<0.001), N stage (*P*<0.001) and M stage (*P*=0.031).

**Table 2:** Analysis of the association between lncRNA AC079630.4 expression and clinicopathological features of lung cancer based on dataset GSE30219.

**Variables**	**Low expression (n=147)**	**High expression (n=146)**	***P* value**
Age (years)	62.5 ± 1.10	63.5 ± 0.77	0.280
Gender			
Male	122 (83.0%)	128 (87.7%)	0.258
Female	25 (17.0%)	18 (12.3%)	
T stage			
T1	59 (41.8%)	107 (73.3%)	<0.001
T2	43 (30.5%)	26 (17.8%)	
T3	22 (15.6%)	9 (6.2%)	
T4	17 (12.1%)	4 (2.7%)	
N stage			
N0	78 (53.8%)	120 (82.2%)	<0.001
N1	34 (23.4%)	19 (13.0%)	
N2	23 (15.9%)	7 (4.8%)	
N3	10 (6.9%)	0 (0.0%)	
M stage			
M0	138 (95.2%)	144 (99.3%)	0.031
M1	7 (4.8%)	1 (0.7%)	

### Association between AC079630.4 expression and lung cancer prognosis

**Figure 4:**
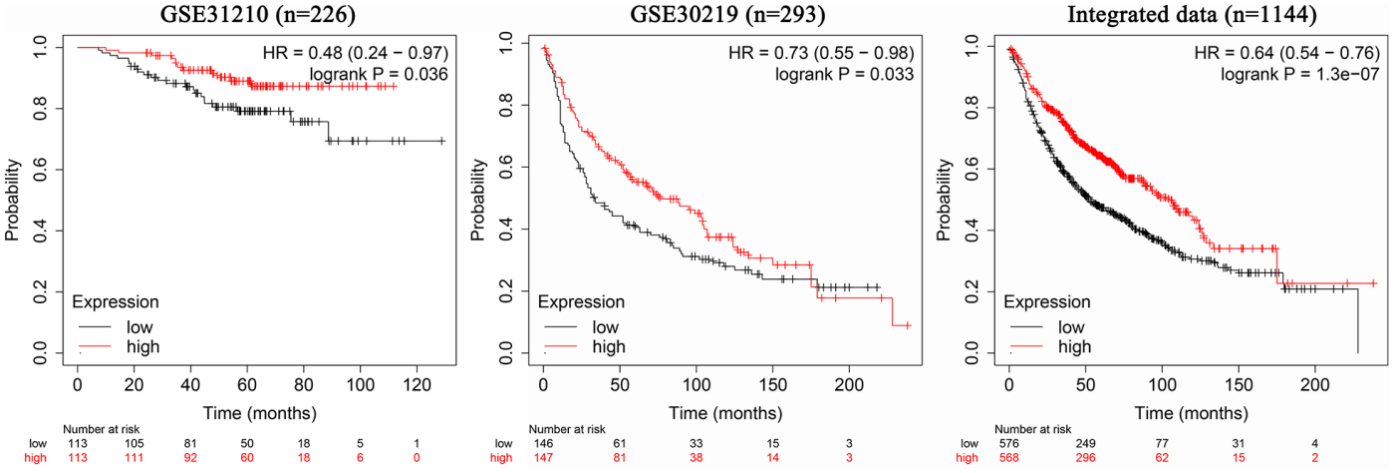
Association between AC079630.4 expression and lung cancer prognosis.

The 226 samples in the dataset GSE31210 were divided into two groups according to the AC079630.4 expression, and the patients with high AC079630.4 expression showed a better prognosis (Hazard ratio (HR): 0.48, 95% confidence interval (CI): 0.24~0.97; *P*=0.036) (Figure 4). In the 293 samples of dataset GSE30219, the patients with high AC079630.4 expression showed a better prognosis (HR: 0.73, 95% confidence interval (CI): 0.55~0.98; *P*=0.033). We also searched the K-M Plotter database which contained 1144 samples from 7 datasets (http://kmplot.com/analysis/index.php), and the patients with high AC079630.4 expression also showed a better prognosis (HR: 0.64, 95% confidence interval (CI): 0.54~0.76; *P*<0.001).

### Potential biological processes associated with AC079630.4

**Figure 5:**
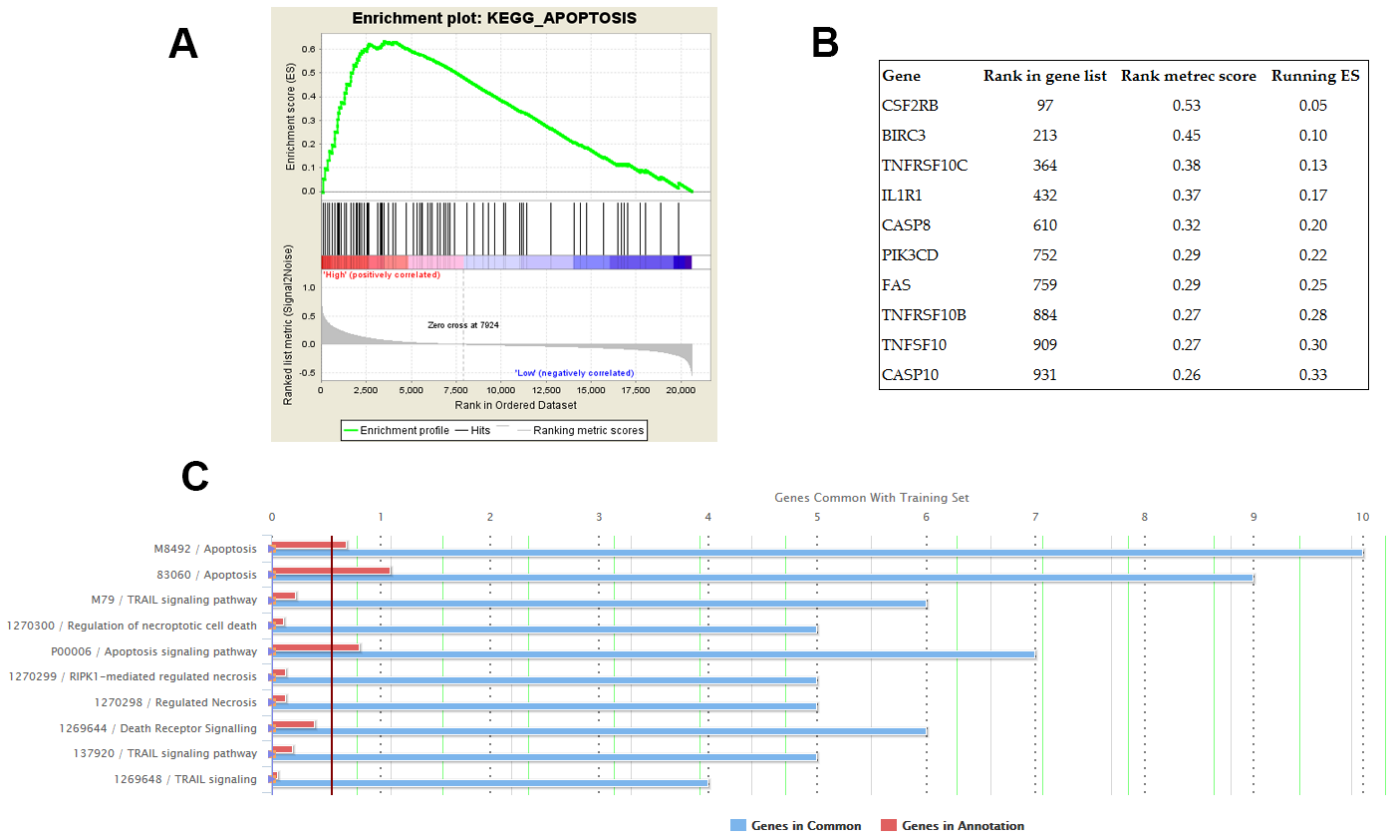
**Potential biological processes associated with AC079630.4.** (**A**) The KEGG pathway of apoptosis was identified by gene set enrichment analysis (GSEA) as the common AC079630.4-related biological pathway based on the datasets of GSE31210 and GSE30219. (**B**) Top 10 apoptosis-enriched genes identified by GSEA. (**C**) Gene ontology (GO) analysis of the top 10 genes.

GSEA was conducted on the datasets of GSE31210 and GSE30219 respectively. The KEGG pathway of apoptosis was identified as the common biological process associated with the genes enriched in the samples with high AC079630.4 expression (Figure 5A). Furthermore, top 10 apoptosis-enriched genes identified by GSEA were chosen for GO analysis (Figure 5B). These genes showed a significant association with apoptosis and TRAIL signaling pathway (Figure 5C).

### Experimental validation

**Figure 6:**
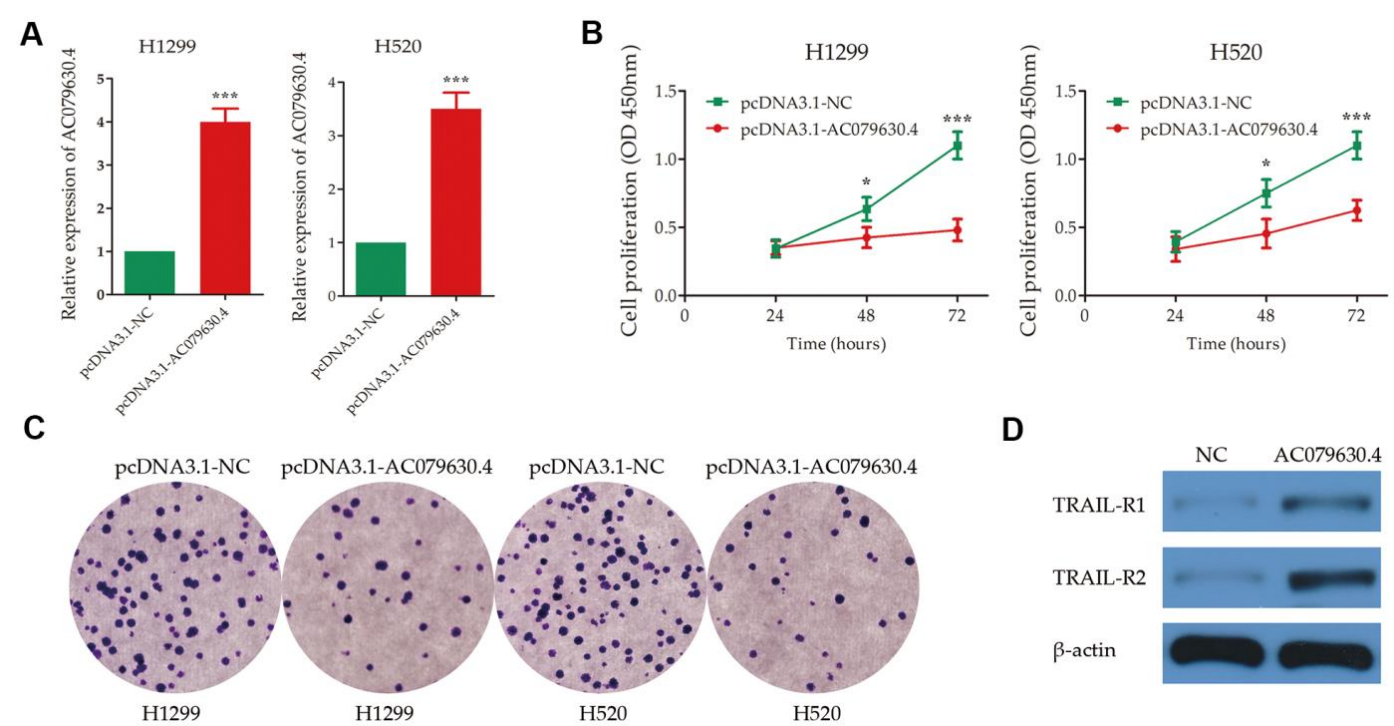
**Experimental validation of the effects of AC079630.4 over-expression on lung cancer cells.** (**A**) Transfection efficiency of AC079630.4 over-expression plasmid. (**B**) Survival curves of transfected cells with AC079630.4 over-expression plasmid (pcDNA3.1-AC079630.4) or the negative control (pcDNA3.1-NC). (**C**) Cell proliferation assay analysis of transfected cells with AC079630.4 over-expression plasmid or the negative control. (**D**) Effect of AC079630.4 over-expression on the expression of TRAIL-R1 and TRAIL-R2. *P<0.05, **P<0.01, ***P<0.001.

After plasmid transfection, the expression of AC079630.4 were up-regulated significantly in the cell lines of both H1299 and H520 (*P*<0.001) (Figure 6A). In cell proliferation assay, the AC079630.4 over-expression group (pcDNA3.1-AC079630.4) had a lower survival rate than the negative control group (pcDNA3.1-NC) (*P*<0.001) (Figure 6B). The over-expression group also had a less number of colonies than the control group (*P*<0.001) (Figure 6C). In the over-expression group, the receptors of TRAIL signaling pathway (TRAIL-R1 and TRAIL-R2) were significantly up-regulated (Figure 6D). These indicated that AC079630.4 over-expression might promote the apoptosis by activating the TRAIL signaling pathway.

To investigate the effects of AC079630.4 over-expression on others apoptotic genes, we also detect the expression of CASP-3 and CASP-8. After AC079630.4 over-expression, both CASP-3 and CASP-8 were up-regulated in HT1299 and HT520 (Supplementary Figure 2). In comparison, CASP-3 and CASP-8 were more significantly up-regulated in HT1299 and HT520 after incubating with 50~100 ng/mL TRAIL for 24h.

## DISCUSSION

In recent year, several studies indicated lncRNAs as robust biomarkers in the diagnosis, treatment and prognosis of multiple carcinomas [11, 12]. To our knowledge, our study found the down-regulation of lncRNA AC079630.4 in lung cancer tissues for the first time. Additionally, patients with low AC079630.4 expression had a more advanced pathological stage and a worse prognosis than those with high expression. The experiment also verified that the over-expression of AC079630.4 could significantly inhibit the proliferation and clonality. These results suggested AC079630.4 as an anti-oncogene.

In mechanism, we found an enrichment of apoptosis-related genes in the samples with high AC079630.4 expression. This was validation in two datasets. Among these related genes, we conducted GO analysis of the top 10 genes. Finally, we found the genes were significantly associated with the TRAIL signaling pathway. In the lung cancer cells with AC079630.4 over-expression, the receptors of TRAIL were up-regulated. These results indicated a potential involvement of AC079630.4 in the biological process of apoptosis through the TRAIL signaling pathway. Previous studies have reported that sensitizing lung cancer cells to TRAIL or cancer cell-expressed TRAIL-R could induce apoptosis and thus attenuate cancer progression, invasion and metastasis [13, 14]. Moreover, the patients with EGRF mutation showed a lower AC079630.4 expression than those without EGRF mutation. In the recent study of Shi et al, the third-generation EGFR inhibitor of osimertinib, selectively and irreversibly inhibits EGFR activating and T790 M mutants, could enhance apoptosis induced by TRAIL primarily in NSCLC with activating EGFR mutations [15]. This indicates the role of EGFR mutations in suppressing TRAIL expression in lung cancer. Our experiments showed that AC079630.4 over-expression could significantly promote TRAIL expression in lung cancer. Thus, there might exist an opposite effect between EGFR mutations and AC079630.4 expression on the regulation of TRAIL in lung cancer.

Furthermore, previous studies have reported that TRAIL signaling pathway could induce cellular apoptosis by up-regulating CASP-3 and CASP-8 [16, 17]. We found that AC079630.4 over-expression could also increase the expression of CASP-3 and CASP-8. Thus, it was unclear whether CASP-3 and CASP-8 were the downstream targets of AC079630.4 or TRAIL signaling pathway. Finally, we found that AC079630.4 might be the upstream regulator of TRAIL signaling pathway, and CASP-3 and CASP-8 might be the downstream targets of TRAIL signaling pathway.

In breast cancer, multiple lncRNAs played an important role in the regulation of TRAIL, like HOTAIR, NEAT1, BCAR4 and DSCAM-AS1 [18]. In hepatocellular carcinoma, the well-established tumor suppressive lncRNA of CASC2 could serve as a sponge of miR-24 and miR-221, and thus modulate TRAIL-induced tumor cell apoptosis [19]. In pancreatic cancer, high HOTAIR levels increased the resistance of pancreatic cancer cells to TRAIL-induced apoptosis via epigenetic regulation of DR5 expression [20]. Furthermore, a piRNA derived from Growth Arrest Specific 5 (GAS5), a tumor-suppressive long non-coding RNA, potently up-regulated the transcription of TRAIL by inducing H3K4 methylation/H3K27 demethylation [21]. These findings indicated an obvious association of lncRNAs in the regulation of TRAIL. In the future, we expected more work in the investigation of the mechanism of this novel lncRNA.

In conclusion, we adopted the methods of bioinformatics analysis and experimental validation, and identified a novel lncRNA of AC079630.4 as a tumor suppressor in lung cancer.

## Supplementary Material

Supplementary Figures

Supplementary Table 1
