# Supplementary material for: LncRNA AC079630.4 expression associated with the progression and prognosis in lung cancer
Source: Aging (Albany NY). 2021 Jul 19;13(14):18658–68. doi: 10.18632/aging.203310 (PMC8351710; doi:10.18632/aging.203310)
Supplement: Supplementary Figures [file aging-13-203310-s001.pdf]

SUPPLEMENTARY FIGURES

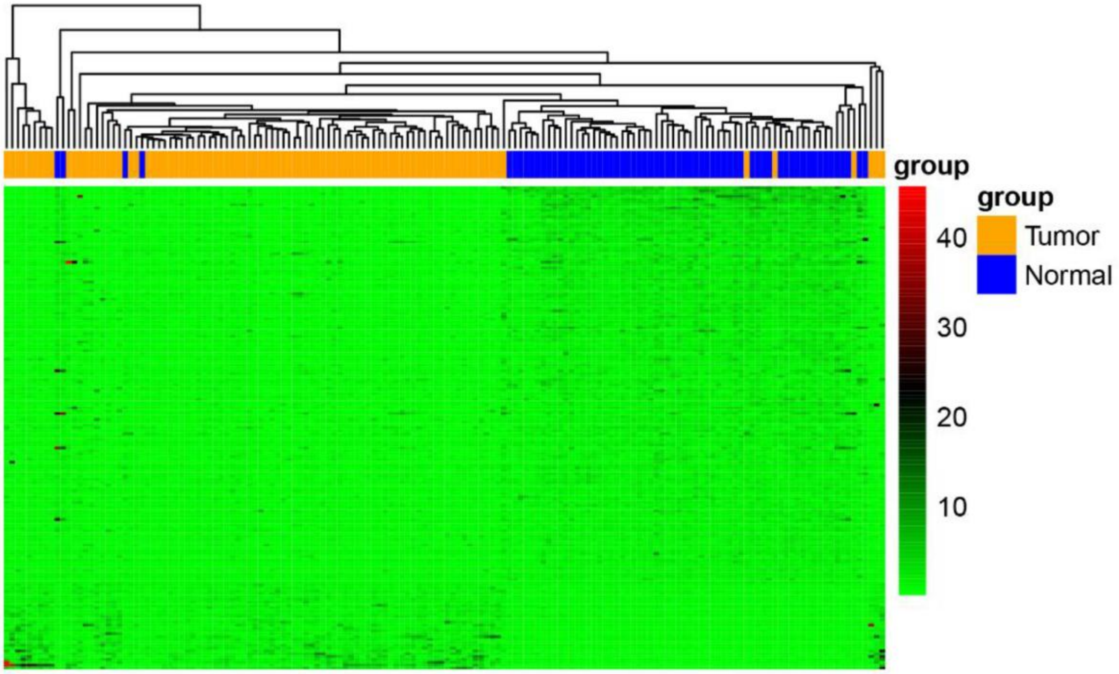

Supplementary Figure 1. Heatmap of the differentially expressed long non-coding RNAs (lncRNAs) in lung cancer (based on the dataset GSE19188).

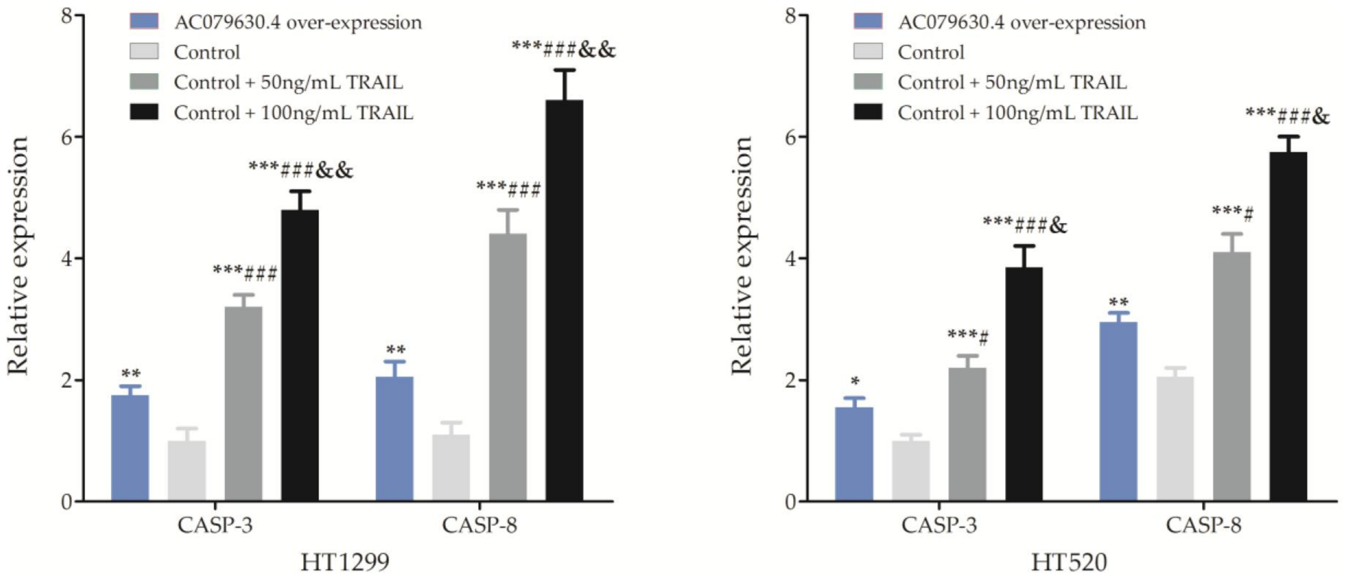

Supplementary Figure 2. The effects of AC079630.4 over-expression and supplementary TRAIL on the expression of CASP-3 and CASP-8 in lung cancer cells. HT1299 and HT520 were treated with AC079630.4 over-expression, and the control with 0~100ng/mL TRAIL for 24h. Compared with the control, *P<0.05, **P<0.01, ***P<0.001. Compared with AC079630.4 over-expression, #P<0.05, ##P<0.01, ###P<0.001. Compared with the control with 50ng/mL TRAIL, &P<0.05, &&P<0.01, &&&P<0.001.
