# Supplementary material for: LncRNA AC079630.4 expression associated with the progression and prognosis in lung cancer
Source: Aging (Albany NY). 2021 Jul 19;13(14):18658–68. doi: 10.18632/aging.203310 (PMC8351710; doi:10.18632/aging.203310)
Supplement: Supplementary Table 1 [file aging-13-203310-s002.docx]

**Supplementary Table 1. List of the differentially expressed long non-coding RNAs (lncRNAs) in lung cancer (based on the dataset GSE19188).**

| **Probe ID** | **Gene symbol** | **Change Fold** | **Adjusted *P* value** |
| --- | --- | --- | --- |
| 228766_at | CD36 | -3.551771847 | 2.68092E-35 |
| 236065_at | AC079630.4 | -2.955949098 | 7.3627E-43 |
| 243818_at | RP1-186E20.2 | -2.869701068 | 1.49748E-39 |
| **1560850_at** | **AC008268.1** | **-2.567084341** | **1.32863E-25** |
| 243813_at | RP11-16M8.2 | -2.451985138 | 3.60925E-38 |
| 239262_at | RP11-736K20.4 | -2.35473529 | 1.16168E-34 |
| 210272_at | CYP2B7P1 | -2.289043288 | 3.83115E-23 |
| 229985_at | BTNL9 | -2.251778313 | 4.75505E-28 |
| 230132_at | AC011526.1 | -2.208270946 | 6.34553E-42 |
| 233947_s_at | RP11-385N17.1 | -2.14565827 | 6.29725E-31 |
| 214696_at | MIR22HG | -1.974593493 | 1.56067E-27 |
| 1556364_at | CTD-2230D16.1 | -1.873647172 | 4.156E-39 |
| 1556314_a_at | RP11-389C8.2 | -1.834370067 | 6.0481E-27 |
| 227554_at | AC004945.2 | -1.80827623 | 8.19321E-30 |
| 229620_at | SEPP1 | -1.744527612 | 2.00508E-26 |
| 1555216_a_at | RP11-332H18.4 | -1.716432527 | 2.32811E-21 |
| 215447_at | AC007319.1 | -1.709806624 | 7.168E-22 |
| 228601_at | HAGLR | -1.657651395 | 1.80941E-20 |
| 220287_at | ADAMTS9 | -1.641319178 | 7.1044E-22 |
| 238720_at | OMG | -1.593270955 | 5.04455E-15 |
| 242340_at | RP1-78O14.1 | -1.588857055 | 2.65864E-30 |
| 213364_s_at | SNX1 | -1.578748806 | 4.62306E-34 |
| 228376_at | GGTA1P | -1.52846477 | 4.54354E-17 |
| 242874_at | RP11-747H7.3 | -1.526409117 | 1.52455E-12 |
| 233289_at | RP4-635A23.6 | -1.518788113 | 4.92895E-24 |
| 215375_x_at | CTD-3222D19.12 | -1.512642144 | 3.37305E-23 |
| 214235_at | CYP3A5 | -1.511644661 | 1.7708E-08 |
| 239919_at | RP11-385N17.1 | -1.510691983 | 8.14247E-29 |
| 1569095_at | RP11-701P16.5 | -1.502765461 | 2.34936E-27 |
| 1557729_at | GRK5 | -1.469613343 | 9.06677E-31 |
| 228391_at | CYP4V2 | -1.468533208 | 6.62319E-20 |
| 227235_at | RP11-588K22.2 | -1.463522594 | 6.42093E-22 |
| 210170_at | PDLIM3 | -1.459502572 | 7.22773E-15 |
| 239847_at | RP11-33E12.2 | -1.45515039 | 1.48654E-18 |
| 232298_at | MBNL1-AS1 | -1.447728406 | 1.96682E-28 |
| 1559605_a_at | AC109642.1 | -1.425209892 | 9.46973E-27 |
| 243023_at | RP11-127B20.2 | -1.413232624 | 5.88766E-22 |
| 243729_at | RP11-747H7.3 | -1.411823581 | 5.54434E-14 |
| 240929_at | CTD-3107M8.4 | -1.39293007 | 4.2049E-25 |
| 201578_at | PODXL | -1.391324805 | 4.2274E-22 |
| 229319_at | RP3-425C14.4 | -1.37877336 | 2.86369E-27 |
| 222312_s_at | RP11-33E12.2 | -1.360817298 | 1.39086E-20 |
| 236632_at | RP11-291L15.2 | -1.320965749 | 1.7095E-23 |
| 1554781_at | CTC-210G5.1 | -1.303232316 | 5.54309E-20 |
| 227917_at | AF228730.14 | -1.29945871 | 3.31933E-21 |
| 244655_at | RP11-327J17.3 | -1.296788431 | 4.70913E-22 |
| 228370_at | SNHG14 | -1.296686529 | 8.46238E-15 |
| 236079_at | RP11-448G15.3 | -1.295170849 | 5.49153E-29 |
| 242290_at | TACC1 | -1.291647238 | 1.57307E-23 |
| 230127_at | RP6-99M1.2 | -1.279999061 | 1.17237E-14 |
| 233591_at | RP11-476D10.1 | -1.267284906 | 1.38858E-20 |
| 236166_at | KIDINS220 | -1.251079542 | 6.01981E-24 |
| 244548_at | CTC-483L24.2 | -1.249869835 | 1.18242E-12 |
| 1559067_a_at | RP11-4O1.2 | -1.226896301 | 1.09811E-20 |
| 233527_at | RP11-677M14.3 | -1.222268889 | 9.30808E-24 |
| 214823_at | XXbac-BPG24O18.1 | -1.222236929 | 1.81201E-15 |
| 242332_at | FENDRR | -1.215018187 | 3.98646E-25 |
| 238081_at | WDFY3-AS2 | -1.210510045 | 5.84769E-24 |
| 1555083_at | RPL13AP17 | -1.207737559 | 2.37582E-22 |
| 228781_at | RP11-541N10.3 | -1.203043148 | 3.05982E-29 |
| 232098_at | DST | -1.196820195 | 1.13028E-11 |
| 207283_at | AC092839.1 | -1.183156715 | 3.18179E-23 |
| 232020_at | SMURF2 | -1.149449054 | 2.16538E-17 |
| 224929_at | TMEM173 | -1.13332193 | 6.38537E-17 |
| 241310_at | NEK5 | -1.122775637 | 3.42912E-11 |
| 238619_at | RP11-138A9.1 | -1.116019646 | 2.28596E-11 |
| 232227_at | RP11-251M1.1 | -1.108591705 | 1.19472E-25 |
| 222348_at | MAST4 | -1.104453277 | 5.3897E-11 |
| 213596_at | CASP4 | -1.10196449 | 6.77205E-15 |
| 229934_at | RP6-159A1.4 | -1.08466636 | 7.72639E-22 |
| 225381_at | MIR100HG | -1.059919705 | 1.1389E-09 |
| 1566482_at | RP11-305O6.3 | -1.050699182 | 9.41548E-14 |
| 1555847_a_at | CTD-3252C9.4 | -1.046330777 | 1.28563E-14 |
| 228647_at | RP11-218M22.1 | -1.039353173 | 1.79513E-13 |
| 235979_at | C7 | -1.031470512 | 7.05882E-13 |
| 236304_at | RP11-67B16.1 | -1.027101555 | 2.53335E-25 |
| 236892_s_at | HOXB-AS3 | -1.025910814 | 2.34978E-11 |
| 239162_at | RP11-40C6.3 | -1.017154512 | 6.67944E-11 |
| 228974_at | ZNF677 | -0.991264419 | 8.44971E-10 |
| 1556003_a_at | RP11-356K23.1 | -0.976773715 | 1.12371E-05 |
| 239656_at | LHFPL3-AS2 | -0.972545397 | 1.24576E-30 |
| 236656_s_at | RP11-490M8.1 | -0.969464907 | 4.18399E-09 |
| 230433_at | RP4-639F20.1 | -0.964126725 | 4.67903E-17 |
| 238893_at | RP11-591N1.1 | -0.959936172 | 1.78811E-14 |
| 223646_s_at | TXLNG2P | -0.959103445 | 1.867E-05 |
| 212225_at | EIF1 | -0.95720088 | 1.57882E-18 |
| 217143_s_at | TRDC | -0.955029119 | 2.33599E-07 |
| 229781_at | AC018647.3 | -0.94828447 | 1.50798E-22 |
| 220936_s_at | H2AFJ | -0.938995035 | 2.8206E-16 |
| 239064_at | RP11-378A13.1 | -0.925388516 | 7.76434E-28 |
| 207474_at | SNRK | -0.914607718 | 2.49593E-13 |
| 1559333_at | AC037193.1 | -0.913325946 | 2.4833E-06 |
| 229699_at | RP11-67L2.2 | -0.910777816 | 1.05537E-11 |
| 1559117_at | RP11-399K21.12 | -0.877289246 | 6.28204E-11 |
| 232632_at | RP11-797A18.5 | -0.87199125 | 1.0859E-08 |
| 243049_at | KB-1396H2.2 | -0.871406714 | 1.49426E-11 |
| 1557359_at | RP1-249H1.4 | -0.869804617 | 5.30867E-13 |
| 225724_at | RP11-349A22.5 | -0.861128331 | 1.84305E-19 |
| 1556732_at | EML4 | -0.856186607 | 2.88692E-12 |
| 235410_at | NPHP3 | -0.853524205 | 2.85059E-12 |
| 217584_at | NPC1 | -0.850387428 | 3.20176E-15 |
| 1558111_at | MBNL1 | -0.84594754 | 6.49602E-08 |
| 217593_at | ZSCAN18 | -0.842165834 | 6.4695E-11 |
| 1557049_at | BTBD19 | -0.840611068 | 1.65987E-13 |
| 236704_at | RP11-326G21.1 | -0.836804471 | 4.11187E-12 |
| 242380_at | AF129075.5 | -0.834977828 | 2.54003E-20 |
| 228710_at | RP11-196G11.5 | -0.82996539 | 4.67415E-16 |
| 215418_at | PARVA | -0.796120359 | 7.87185E-17 |
| 214829_at | AASS | -0.784800019 | 1.01783E-09 |
| 1559882_at | SAMHD1 | -0.774929008 | 1.01092E-08 |
| 207063_at | NCRNA00185 | -0.774161237 | 3.87596E-11 |
| 239580_at | RP11-588K22.2 | -0.77407909 | 2.79386E-12 |
| 229809_at | POU6F1 | -0.77237475 | 8.87208E-23 |
| 1557553_at | PPP1R12B | -0.771690387 | 5.64793E-07 |
| 236996_at | RP11-295M18.6 | -0.771065261 | 4.14665E-21 |
| 236396_at | RP11-522B15.3 | -0.768652715 | 7.21322E-19 |
| 237168_at | NEK5 | -0.760174313 | 1.32073E-08 |
| 232425_at | SNX25 | -0.756092586 | 5.13123E-25 |
| 233480_at | TMEM43 | -0.752654667 | 5.3898E-10 |
| 230937_at | XXbac-BPG181B23.7 | -0.749596748 | 3.03707E-11 |
| 229130_at | RP11-774O3.3 | -0.749156034 | 1.58849E-09 |
| 241425_at | NUP58 | -0.745474986 | 1.46231E-12 |
| 235362_at | RP4-639F20.1 | -0.738460015 | 2.05673E-12 |
| 243555_at | RP11-752L20.3 | -0.734813839 | 3.98954E-12 |
| 1554786_at | CASS4 | -0.732054605 | 2.55212E-20 |
| 242358_at | RP11-877E17.2 | -0.718405235 | 6.13181E-09 |
| 228722_at | PRMT2 | -0.716842889 | 1.09031E-12 |
| 236118_at | RP11-627G18.3 | -0.706187343 | 5.92016E-16 |
| 1553448_at | RP11-544L8__B.4 | -0.68851899 | 3.57186E-15 |
| 229455_at | AC083843.1 | -0.685520849 | 1.55506E-05 |
| 1560128_x_at | AC116366.6 | -0.678982167 | 9.92943E-14 |
| 243974_at | RP3-331H24.6 | -0.672734133 | 6.14078E-15 |
| 232049_at | CTB-36H16.2 | -0.660683656 | 3.94991E-24 |
| 240421_x_at | SAV1 | -0.660672478 | 2.20028E-16 |
| 228160_at | RP11-835E18.2 | -0.651984777 | 8.8824E-07 |
| 232715_at | RP11-1024P17.1 | -0.65113214 | 7.06713E-33 |
| 238652_at | KDM1A | -0.626413882 | 1.53195E-15 |
| 241369_at | RP11-14D22.5 | -0.614731099 | 8.71203E-15 |
| 232833_at | RP6-201G10.2 | -0.608624643 | 1.14642E-18 |
| 237005_at | AC034193.5 | 0.638044398 | 1.19801E-11 |
| 230294_at | RP5-1125A11.1 | 0.67484213 | 2.18715E-11 |
| 222214_at | CTD-2349P21.3 | 0.711244808 | 1.13979E-09 |
| 237943_at | TMCC1 | 0.717213778 | 6.1714E-09 |
| 1568857_a_at | NBR1 | 0.735413619 | 1.34647E-13 |
| 1561689_at | RP11-258C19.5 | 0.767202447 | 2.51178E-16 |
| 233436_at | MTBP | 0.783286161 | 8.2656E-15 |
| 236798_at | RP11-393B14.1 | 0.793909131 | 3.68434E-08 |
| 230097_at | GART | 0.843905669 | 4.50613E-11 |
| 228357_at | UNK | 0.84421217 | 2.4976E-12 |
| 1556103_at | RP11-480A16.2 | 0.855110315 | 7.4601E-17 |
| 227517_s_at | GAS5 | 0.882115212 | 3.58225E-10 |
| 224185_at | RP11-199F11.2 | 0.900544258 | 3.0529E-15 |
| 239657_x_at | FOXO6 | 0.919817182 | 5.34356E-14 |
| 238727_at | RP11-384O8.1 | 0.963172144 | 4.96832E-08 |
| 240616_at | RP11-12G12.7 | 0.991367593 | 1.33003E-14 |
| 229519_at | FXR1 | 1.063865363 | 8.73165E-17 |
| 240382_at | RP3-512B11.3 | 1.131094729 | 7.40832E-16 |
| 228564_at | AC017048.3 | 1.131772822 | 1.83717E-09 |
| 231233_at | RP11-480I12.3 | 1.192844762 | 2.95774E-19 |
| 1558290_a_at | PVT1 | 1.274194707 | 3.15963E-17 |
| 227249_at | CTB-193M12.5 | 1.290613042 | 7.45513E-14 |
| 241763_s_at | FBXO32 | 1.361462194 | 2.35913E-22 |
| 213248_at | RP1-152L7.5 | 1.434760664 | 1.0052E-12 |
| 237563_s_at | RP11-295G20.2 | 1.444649973 | 7.85775E-16 |
| 238632_at | RP11-44F21.5 | 1.448600864 | 8.17647E-12 |
| 243018_at | RP11-1L12.3 | 1.460739691 | 1.93887E-14 |
| 214073_at | CTTN | 1.761531939 | 3.97042E-19 |
| 232202_at | RP3-523K23.2 | 1.969005812 | 2.09841E-12 |
| 227452_at | LINC00511 | 2.338433138 | 3.86512E-24 |
